# Micropulse Laser Therapy in Central Serous Chorioretinopathy

**DOI:** 10.3390/clinpract14060194

**Published:** 2024-11-14

**Authors:** Flaviu Bodea, Andrei-Flavius Radu, Ruxandra-Florina Bodog, Teodora Maria Bodog, Cristina Ariadna Nicula

**Affiliations:** 1 Doctoral School of Biomedical Sciences, University of Oradea, 410087 Oradea, Romania; bodea.flaviusionut@didactic.uoradea.ro (F.B.); bodog.teodoramaria@student.uoradea.ro (T.M.B.); nicula.cristina@didactic.uoradea.ro (C.A.N.); 2 Department of Preclinical Disciplines, Faculty of Medicine and Pharmacy, University of Oradea, 410073 Oradea, Romania; 3 Department of Oro-Maxilo Facial Surgery and Radiology, Faculty of Medicine, University of Medicine and Pharmacy “Iuliu Hațieganu”, 400012 Cluj Napoca, Romania

**Keywords:** micropulse laser therapy (MPLT), central serous chorioretinopathy (CSCR), corticosteroid treatment, subretinal fluid

## Abstract

**Background**: Central serous chorioretinopathy (CSCR) is a retinal condition characterized by the accumulation of subretinal fluid, often linked to elevated levels of endogenous corticosteroids and stress-related hormones, which can lead to visual disturbances. This connection may explain the association of CSCR with high stress levels and the use of corticosteroid medications. Although many cases resolve spontaneously, persistent or severe instances may require intervention. **Case Description**: Our report presents a case of acute CSCR in a 33-year-old male who developed the condition following corticosteroid treatment for acute parotiditis and left submandibular lymphadenopathy. Initial presentation was 6 weeks after corticosteroid treatment was initiated. Diagnostic evaluation with optical coherence tomography (OCT) and fluorescein angiography confirmed the presence of subretinal fluid at the retinal pigment epithelium–Bruch’s membrane complex. Micropulse laser therapy (MPLT) was applied to address the leakage points, leading to significant fluid reduction at a two-week follow-up. By six weeks, the complete resolution of subretinal fluid was documented, with substantial visual recovery. **Conclusions**: This case confirms MPLT as an effective treatment for CSCR, particularly when conservative management is insufficient. Unlike traditional photocoagulation, MPLT offers a safer alternative, minimizing the risk of retinal damage, such as permanent scotomas. **Clinical Significance**: This case underscores the importance of carefully monitoring patients undergoing corticosteroid therapy for potential ocular complications and highlights the role of MPLT as a safe and effective option for managing persistent CSCR, protecting the surrounding retinal tissue from damage while achieving significant therapeutic outcomes.

## 1. Introduction

Ophthalmic disorders encompass a wide variety of conditions, some of which specifically involve the retina and its supporting structures [1]. One such condition is central serous chorioretinopathy (CSCR), which represents a limited serous detachment of the neurosensory retina, changes usually confined to the macula, associated with leakage of fluid from the altered retinal pigment epithelium (RPE) [2,3]. In 1866, it was described by Albrecht von Graefe as a recurrent central retinitis [4]. It mainly affects men in their 30s to 40s, type A personalities, with a ratio of male to female of 8:1 [5,6,7,8,9]. CSCR is characterized by decreased visual acuity, distorted vision, blurred vision, a relative central scotoma, metamorphopsia, reduced contrast sensitivity, and mild dyschromatopsia. CSCR tends to appear usually unilateral, but in elderly patients, bilateral affection was noted more often [10].

There are two forms of CSCR, an acute and a chronic one. Chronic CSCR does not have a generally accepted duration, but some of the authors speak of chronic CSCR if the symptoms last more than 3 months, while other authors are considering CSCR as being chronic if the symptoms are present for more than 6 months [11]. Acute CSCR is characterized by monofocal or paucifocal RPE changes that tend to resolve spontaneously. Meanwhile, the chronic form of CSCR means that there is multifocal or diffuse RPE depigmentation associated with a serous detachment of the retina [12].

The treatment for both acute and chronic CSCR can include one of the following options: observation for spontaneous recovery, focal laser, photodynamic laser therapy, eplerenone drug therapy, and intravitreal anti-vascular endothelial growth factor. One of the newest options available is stimulation of the RPE with subthreshold micropulse laser. This treatment stimulates the RPE cells, correcting the pump function and improving the outer blood–retina barrier. After applying the laser spots, there is no visible scar, because the laser energy that arrives at the tissue is in the form of short pulses and does not result in coagulation or scaring as a conventional laser does [13].

After more than a decade and hundreds of interventions, we believe that the time has come to present a small part of the experience gained over the years. This paper presents a case of acute CSCR that developed as a complication following corticosteroid therapy administered for the treatment of acute parotiditis. This case highlights the potential ocular side effects associated with systemic corticosteroid use, particularly the onset of CSCR, and the need for careful consideration of corticosteroid use. Also, interdisciplinary collaboration between specialists and the importance of timely intervention in the management of associated complications (such as CSCR) also have their well-established place.

## 2. Case Presentation

A 33-year-old male presented to the ophthalmology department of the Emergency Clinical County Hospital in Oradea, Romania with a 3-week history of insidious vision alteration in his left eye, with presumptive diagnosis of macular edema received 2 weeks before, for which he was referred to have further investigations.

His medical history indicated good overall health, aside from the recent episode of left acute parotiditis and left submandibular lymphadenopathy, for which he had been treated with prednisone 5 mg (3 × 1 tb/day for 10 days, followed by 2 × 1 tb/day for 3 days and a subsequent 1 tb/day for one day) by the ear, nose, and throat (ENT) department. 

Physical examination was normal with resolution of parotiditis symptoms at first ophthalmological presentation. The patient had a normal body weight, with no history of smoking, alcohol consumption, or drug use.

Specific ophthalmological examination began with measurement of visual acuity using the Topcon CC-100 visual chart, which revealed normal visual acuity of 1.0 (logMAR 0) in both eyes. However, the patient reported positive dysphotopsia, metamorphopsia, and subjective blurry vision in the left eye. Slit-lamp examination (SL-D4, Topcon Corporation, Tokyo, Japan) revealed a normal anterior pole, including the conjunctiva, cornea, anterior chamber, and lens. Pupillary reflexes and pupil diameter were also normal.

**Figure 1:**
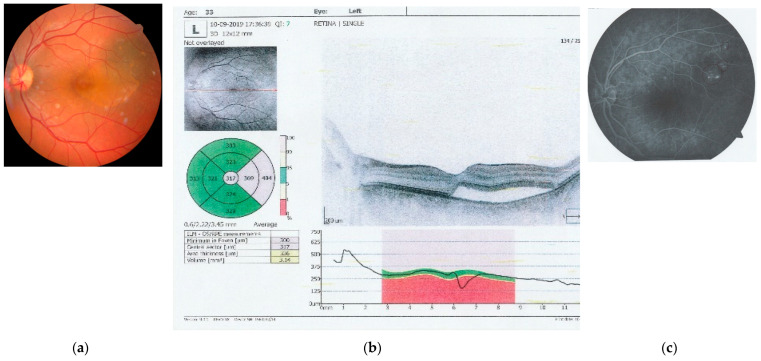
Images captured at initial investigations: (**a**) left eye retinal photography that captures the optic nerve, central retinal artery and vein, and the macula, with alteration of the color and profile of the macular central and temporal region (auto fundus camera -Nidek AFC-330, Japan, after pharmacological dilation of the pupil with tropicamide 1%); (**b**) OCT image of the macula of the left eye, showing a neuro-sensory retinal detachment of the fovea and temporal macula and the presence of subretinal fluid; and (**c**) angio fluorography of the left eye, obtained by IV administration of fluorescein, showing leakage points in the macular region, supero-temporal from the fovea (AFG equipment Carl Zeiss Meditech, Germany).

Examination of the posterior pole using a Volk Digital Wide-Field Lens (Volk Optical, Mentor, OH, USA) showed normal findings in the right eye. In the left eye, the optic nerve was well-contoured with normal color and excavation. However, the macular area revealed a round, well-defined lesion with altered color, indicating the presence of subretinal fluid underneath the neurosensory retina, with the absence of the usual light reflex at the umbo. The retinal vasculature (central retinal artery and vein with their branches) and periphery were normal (Figure 1a). It must be also mentioned that all the pictures are from the personal archive of the first author.

Further investigations were performed to better characterize the lesion. Optical coherence tomography (OCT) (Optopol Technology, Zawiercie, Poland) of the macula confirmed the presence of neurosensory retinal detachment in the foveal and parafoveal temporal regions, with subretinal fluid accumulation (Figure 1b). Fluorescein angiography (FA) (Zeiss Meditec, Jena, Germany) revealed multiple focal leakage points from the superior choroid, with pooling of the fluorescein dye, indicating disruption of the external blood–retinal barrier at the Bruch’s membrane–RPE interface (Figure 1c).

Based on these findings, a diagnosis of unilateral acute CSCR in the left eye was established. After obtaining informed consent, subthreshold micropulse laser therapy (SMPLT) was performed on the retinal areas showing leakage on the FA. The IQ 577 laser (Iridex, Mountain View, CA, USA) was used in micropulse mode, which is a fovea-friendly yellow laser with minimal absorption by the macular xanthophyll pigment, thereby protecting the central macula from excessive thermal energy and subsequent coagulation of proteins. A total of 91 micropulses were applied over three leakage areas using a setting of 240 mW energy, 200 µm spot diameter, and 200 ms pulse duration, with a Volk H-R Area Centralis treatment lens. The duty cycle of the micropulsed laser for retinal application was set at 5%, indicating that the laser remained active for only 5% of each micropulse duration.

**Figure 2:**
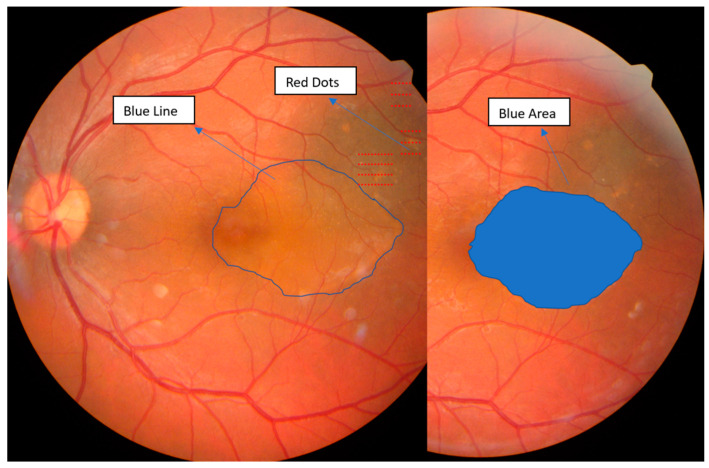
Fundus retino-photography of the left eye, illustrating with red dots, the area of the laser treatment over the leakage points, with the blue line, the initial fluid area in the macular region, and with the blue area, the reduction of the fluid area at two weeks. Images obtained with auto fundus camera (Nidek AFC-330, Gamagori, Japan).

**Figure 3:**
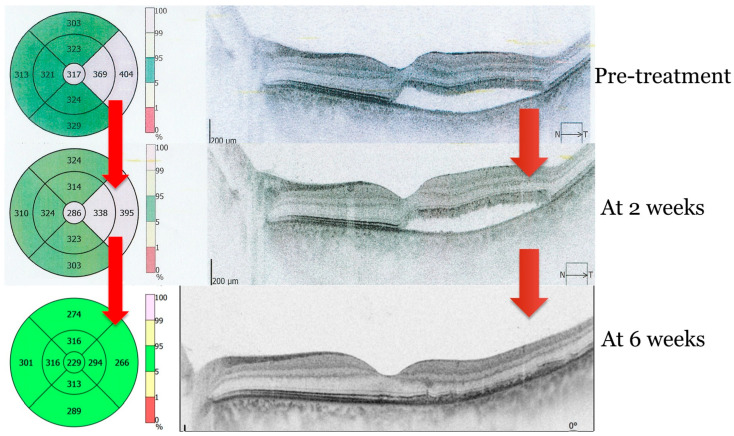
OCT image of subretinal fluid resolution. Sequences before and after subthreshold micropulse laser therapy (SMPLT) at 2 and 6 weeks, respectively. Image obtained with OPTOPOL REVO NX (Optopol Technology, Zawiercie, Poland).

Follow-up evaluations at two and six weeks showed marked improvement, with progressive reabsorption of subretinal fluid and complete retinal reattachment by six weeks. Figure 2 depicts the evolution of subretinal fluid over a two-week period, highlighting the gradual decrease in fluid accumulation and associated improvements in retinal morphology and function. Overall, visual quality showed marked improvement, with complete resolution of symptoms as shown in Figure 2 and Figure 3.

## 3. Discussion

Conservative management, including observation, is often the first-line approach in managing acute CSCR, as many cases resolve spontaneously. However, the optimal timing of intervention remains a subject of debate due to the availability of new treatment options and the need for a more patient-centered approach [14,15]. While spontaneous remission is possible, delaying treatment can lead to prolonged visual impairment, patient distress, and potential chronicity of the condition [16]. 

While photodynamic therapy has demonstrated greater efficacy over micropulse laser for treating CSR, the potential utility of micropulse laser should not be overlooked, particularly given the current shortage of verteporfin [17].

Micropulsed laser treatment (MPLT) is generally recommended after the first month of disease progression. However, in certain cases, it may be beneficial to apply it earlier, particularly for patients who require excellent visual acuity for their work [18,19].

For the case presented in this report, the decision was to apply MPLT, after three weeks from the onset of symptoms. This form of subthreshold laser therapy uses short, low-energy laser pulses, which minimize thermal damage to retinal tissues while stimulating the RPE to pump fluid away from the subretinal space [20]. 

The proven safety of SMPLT allows for the consideration of earlier treatment of patients with CSCR. Recent studies demonstrated that patients with CSCR of less than six months’ duration who received MPLT achieved better functional outcomes with early application of this procedure [14,15]. 

Unlike traditional photocoagulative laser treatment, which can cause retinal scarring and permanent visual defects, MPLT does not induce thermal injury, allowing for safe application even in sensitive areas such as the macula [21]. In this case, the use of MPLT resulted in the significant reduction of subretinal fluid within two weeks and the complete resolution at six weeks, without causing any damage to the retina or other adverse effects.

In the management of CSCR, complications such as focal hyper- or hypo-pigmentation, atrophy or hyperplasia of the RPE, and the development of choroidal neovascularization (CNV) present significant challenges [22]. Studies have shown that CNV can arise either as part of the natural progression of chronic CSCR or as a complication of focal treatments, with reported prevalence rates ranging from 2% to 15.6% [23]. Considering these risks, the choice of treatment for CSCR must be carefully considered.

MPLT has been shown to reduce central retinal thickness (CRT) and improve visual acuity without damaging retinal tissue. In this case, the 577 nm yellow laser, known for its poor absorption by the macular xanthophyll pigment, was used safely over the fovea, protecting delicate retinal structures while resolving subretinal fluid [24,25].

This case highlights the importance of early diagnosis and timely intervention in CSCR, particularly in patients with known risk factors, such as corticosteroid use. While conservative management remains appropriate for many cases, prolonged observation without active intervention may lead to sustained retinal damage in more persistent cases. MPLT provides an effective and safe alternative to more invasive procedures. However, further research is needed to explore the long-term outcomes of MPLT in CSCR, particularly in comparison to other treatment modalities such as photodynamic therapy (PDT) and anti-VEGF injections. 

## 4. Clinical Significance

This case emphasizes the need for vigilant monitoring of patients undergoing corticosteroid therapy, given the potential for ocular complications such as CSCR. It also highlights the effectiveness of SMPLT as a safe and efficient option for managing early cases of CSCR. Initiating treatment early is crucial in acute CSCR to protect retinal photoreceptor cells from prolonged exposure to subretinal fluid, which can lead to lasting damage. Therefore, SMPLT stands as one of the most effective and safest treatment options in the management of acute CSCR, offering therapeutic efficacy without the risks of retinal scarring or vision loss.

## 5. Conclusions

This case demonstrates the successful use of MPLT in the treatment of acute CSCR after corticosteroid treatment. The therapy’s ability to resolve subretinal fluid without causing retinal damage makes it a valuable addition to the treatment options for CSCR. This case also highlights the importance of individualized treatment approaches and the need for continued research into the optimal management of CSCR.

## Data Availability

Data supporting the reported results and the image archive are available from the first author. Also, some data of the patient is unavailable due to ethical restrictions.
